# Molecular Immune Responses to Aerosol Challenge with *Francisella tularensis* in Mice Inoculated with Live Vaccine Candidates of Varying Efficacy

**DOI:** 10.1371/journal.pone.0013349

**Published:** 2010-10-12

**Authors:** Hua Shen, Gregory Harris, Wangxue Chen, Anders Sjostedt, Patrik Ryden, Wayne Conlan

**Affiliations:** 1 Institute for Biological Sciences, National Research Council Canada, Ottawa, Ontario, Canada; 2 Department of Clinical Microbiology, Clinical Bacteriology, Umeå University, Umeå, Sweden; 3 Department of Mathematics and Mathematical Statistics, Department of Statistics, Computational Life Science Cluster, Umeå University, Umeå, Sweden; Louisiana State University, United States of America

## Abstract

**Background:**

*Francisella tularensis* is a facultative intracellular bacterial pathogen and the etiological agent of tularemia. The subspecies *F. tularensis tularensis* is especially virulent for humans when inhaled and respiratory tularemia is associated with high mortality if not promptly treated. A live vaccine strain (LVS) derived from the less virulent *holarctica* subspecies confers incomplete protection against aerosol challenge with subsp. *tularensis*. Moreover, correlates of protection have not been established for LVS.

**Methodology/Principal Findings:**

In the present study we compare molecular immune responses elicited by LVS and two defined deletion mutants of clinical subsp. *tularensis* strain, SCHU S4, that confer enhanced protection in a mouse model. BALB/c mice were immunized intradermally then challenged with an aerosol of SCHU S4 six weeks later. Changes in the levels of a selected panel of cytokines and chemokines were examined in the lungs, spleens, and sera of vaccinated and challenged mice. Mostly, increased cytokine and chemokine levels correlated with increased bacterial burden. However, after adjusting for this variable, immunization with either of the two Schu S4 mutants resulted in higher levels of several pulmonary cytokines, versus those resulting after LVS immunization, including IL-17. Moreover, treatment of mice immunized with *ΔclpB* with anti-IL-17 antibodies post-challenge enhanced lung infection.

**Conclusions/Significance:**

This is the first report characterizing local and systemic cytokine and chemokine responses in mice immunized with vaccines with different efficacies against aerosol challenge with virulent *F. tularensis* subsp. *tularensis*. It shows that increases in the levels of most of these immunomodulators, including those known to be critical for protective immunity, do not superficially correlate with protection unless adjusted for the effects of bacterial burden. Additionally, several cytokines were selectively suppressed in the lungs of naïve mice, suggesting that one mechanism of vaccine action is to overcome this pathogen-induced immunosuppression.

## Introduction

Typhoidal tularemia initiated by inhalation of *Francisella tularensis* subsp. *tularensis* had an historical mortality rate of >50% prior to the advent of chemotherapy [1]. This coupled with its low inhaled infectious dose (10 CFU or less) led to *F. tularensis* subsp. *tularensis* being developed as an aerosolizable biological warfare agent during World War II and the Cold War. To counter this threat, the US Army developed a live vaccine strain, *F. tularensis* LVS, from an attenuated Russian strain derived from the less virulent subsp. *holarctica* [1]. In human volunteer studies LVS administered by scarification was shown to be 25–100% effective against aerosol challenge and 66–90% effective against intradermal (ID) challenge with *F. tularensis* subsp. *tularensis* strain SCHU S4 [2], [3], [4]. Efficacy appeared to be dependent on the vaccine and challenge doses, and on the intervening period between vaccination and challenge. Efficacy was defined as an absence of disease symptoms following challenge, the appearance of which automatically led to treatment with antibiotics. Hence, many of the adjudged vaccine failures in these studies might well have survived an otherwise lethal challenge. Serological assays on volunteers post-vaccination, failed to establish a correlation between antibody agglutination titre and protection from disease symptoms [5]. The absence of a correlate of protection, coupled with regulatory concerns over its method of manufacture and attenuation has prevented the licensure of LVS for general use. Modern technical approaches might be able to overcome these issues.

Nevertheless, it might be possible to make an improved novel vaccine, especially against airborne challenge. To this end we have been investigating the utility of well-defined deletion mutants of SCHU S4 to serve this purpose, not least because they express antigens unique to subsp. *tularensis* that might contribute significantly to protective immunity. However, due to the dearth of natural human tularemia infections initiated by inhaling subsp. *tularensis*, novel vaccines will need to be tested according to the FDA Animal Rule to gain regulatory approval (http://www.fda.gov/cber/rules/humeffic.htm). The Animal Rule allows for efficacy testing of a vaccine to be performed exclusively using relevant animal models of tularemia provided that its mechanism of action in such models predicts its efficacy in humans. Given that protection itself remains the sole evidence of LVS efficacy in humans, it seems reasonable to begin looking for correlates of protection in vaccinated and challenged hosts, in the hope that this will indicate candidate correlates in unchallenged vaccinees.

Mouse models have been the mainstay of *F. tularensis* infection and immunity research for the past quarter century [6]. The major perceived weakness of these models is their relatively high susceptibility to airborne *versus* systemic challenge with subsp. *tularensis* following systemic immunization with LVS. However, this provides a sensitive means for comparing the relative efficacy against inhalation tularemia of novel vaccines *versus* LVS, the current gold standard. In mice, LVS-elicited immunity to systemic or respiratory challenge with subsp. *tularensis* is known to be critically dependent on the actions of interferon gamma (IFNγ) and CD4^+^ and CD8^+^ T cells [7], [8], [9]. *Francisella*-specific CD4^+^ and CD8^+^ T cells persist for prolonged periods in the circulation of humans immunized with LVS or convalescent patients, and secrete IFNγ when re-stimulated with specific antigens [10], [11], [12], [13], [14], [15]. Additionally, early molecular and cellular events following LVS vaccination of humans has recently been documented [16], [17]. However, none of the aforementioned immune responses have been correlated to protection in humans since no challenge studies were performed. In this regard, LVS is effective in BALB/c, but not C57BL/6 mice despite the fact that both generate pathogen-specific cell-mediated immune responses following vaccination [8], [18], [19], [20]. Thus, the mere generation of IFNγ-producing T cells appears to be an insufficient criterion for predicting LVS efficacy in mice, and possibly humans.

Recently, we showed that mutant strains of SCHU S4, missing either the *clpB* gene or both the *fupA* (*FTT0918*) and *capB* genes were at least as attenuated as LVS, but afforded better protection against an aerosol challenge with wild-type SCHU S4 in a murine model, significantly so in the case of *ΔclpB* [21]. In the present study we have vaccinated mice with either of these mutants or LVS and compared a selection of molecular immune responses in the lungs, spleen, and serum following aerosol challenge with SCHU S4 in an effort to reveal potential correlates of protection. The results showed that mice immunized with either of the SCHU S4-based mutants produced significantly more IL-17 and contained fewer bacteria in the lungs than mice immunized with LVS by day 7 of infection. No other cytokine or chemokine showed such a straightforward inverse correlation with lung bacterial burden. However, when the latter variable was adjusted for, several other cytokines and chemokines were expressed at relatively higher levels in mice immunized with *ΔclpB* or *ΔfupAΔcapB*. Treating immunized mice with anti-IL-17 mabs, reduced pulmonary IL-17 levels and increased the lung bacterial burden. No useful correlates of protection were observed in the serum. Compared to immunized mice, several lung cytokine responses appeared to be suppressed in naïve mice, whereas in the serum many of the same molecules were found at higher levels in the latter versus former mice. The consequences of these findings for future vaccine development are discussed.

## Results

### Efficacy of SCHU S4 mutants versus LVS against aerosol challenge

**Figure 1:**
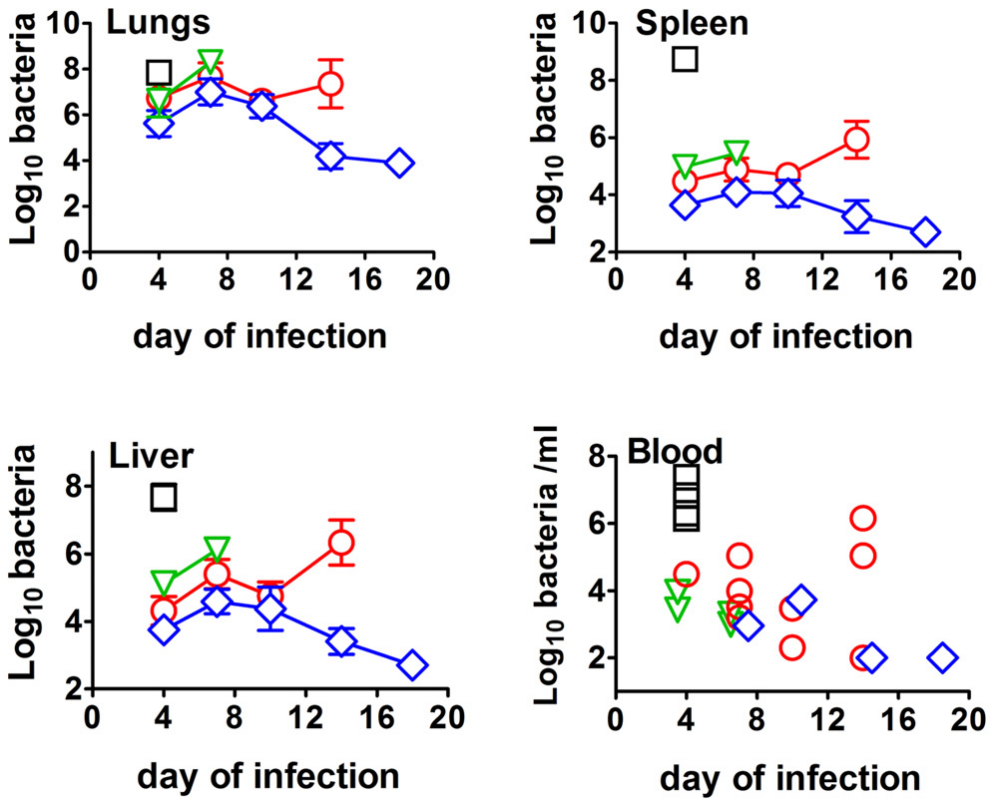
Course of infection in mice immunized ID with 10^5^ CFU of SCHU S4-based mutants *ΔfupAΔcapB* (red), ΔclpB (blue), LVS (green) and in naïve mice (black) following aerosol challenge at six weeks post vaccination.

Throughout the results section all references to differences being significant indicate statistically significant differences at P<0.05 or P<0.0045 (to account for mass significance) by the tests stated in materials and methods. Previously we showed that mice immunized ID with 10^5^ CFU of *ΔclpB* were significantly better protected than LVS immunized mice against a subsequent aerosol challenge with SCHU S4 [21]. Protection in mice immunized with *ΔfupAΔcapB* was intermediate between the former two groups, but this was not significant in either comparison. In the present study, similarly immunized mice were challenged along with naïve mice six weeks later with a low dose aerosol of SCHU S4. Surviving mice were killed on the stated days post-infection for bacteriology. The course of infection in each of the four groups is shown by figure 1. By day 4 of infection, all immunized groups harbored significantly fewer bacteria in their lungs than naïve mice, and lung burdens in mice immunized with *ΔclpB* were significantly lower than in mice immunized with *ΔfupAΔcapB.* All vaccinated groups contained significantly fewer bacteria than naive mice in their livers and spleens at this time. The liver and spleen burden in mice immunized with *ΔclpB* was significantly lower than in mice immunized with LVS at this time. All remaining naïve mice were dead by day 5 of infection. By day 7 of challenge, mice immunized with *ΔclpB* contained significantly fewer bacteria in all organs compared to mice immunized with LVS. None of the latter mice survived to day 10 by which time there was no significant difference in the bacterial burdens in mice immunized with *ΔfupAΔcapB* or *ΔclpB*. Between days 7–10, bacterial burdens decreased somewhat in the organs of both groups. However, between days 10–14, bacterial burdens increased in all organs in mice immunized with *ΔfupAΔcapB*, but decreased further in all organs in mice immunized with *ΔclpB*, and the burdens in each organ were significantly different between these two groups at this time. No mice immunized with *ΔfupAΔcapB* survived to day 18, by which time surviving mice immunized with *ΔclpB* were continuing to resolve infection in all organs. All groups of immunized mice controlled to varying degrees the massive bacteremia associated with progressive primary tularemia. We have obtained similar results previously [21].

**Figure 2:**
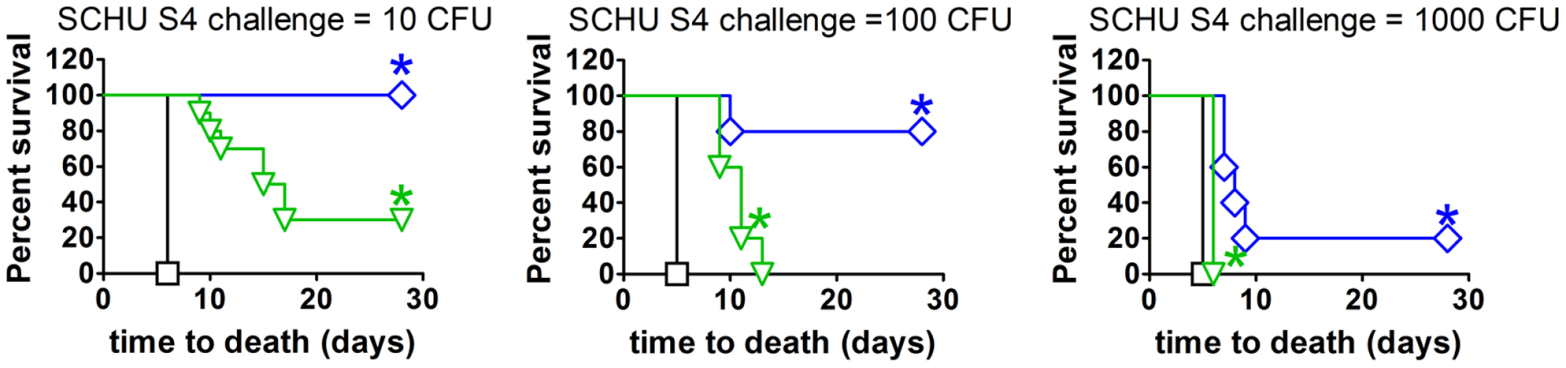
Survival of control mice (black) or mice immunized ID with LVS (green) or *ΔclpB* (blue) following IN challenge with different doses of SCHU S4 six weeks post vaccination. Blue asterisk, significantly greater survival than control mice or mice immunized with LVS; green asterisk, significantly greater survival than control mice.

Additional experiments showed that mice immunized ID with either LVS or *clpB* survived ID challenge with 100,000 CFU of SCHU S4 (not shown). However, the latter mice were significantly better protected than LVS-immunized mice against IN challenge with 10, 100, or 1000 CFU of SCHU S4 (figure 2). The IN route was chosen for this experiment because it allowed more precision with the delivered doses of SCHU S4 than did aerosol exposure.

### Changes in lung cytokine and chemokine levels post-challenge

**Figure 3:**
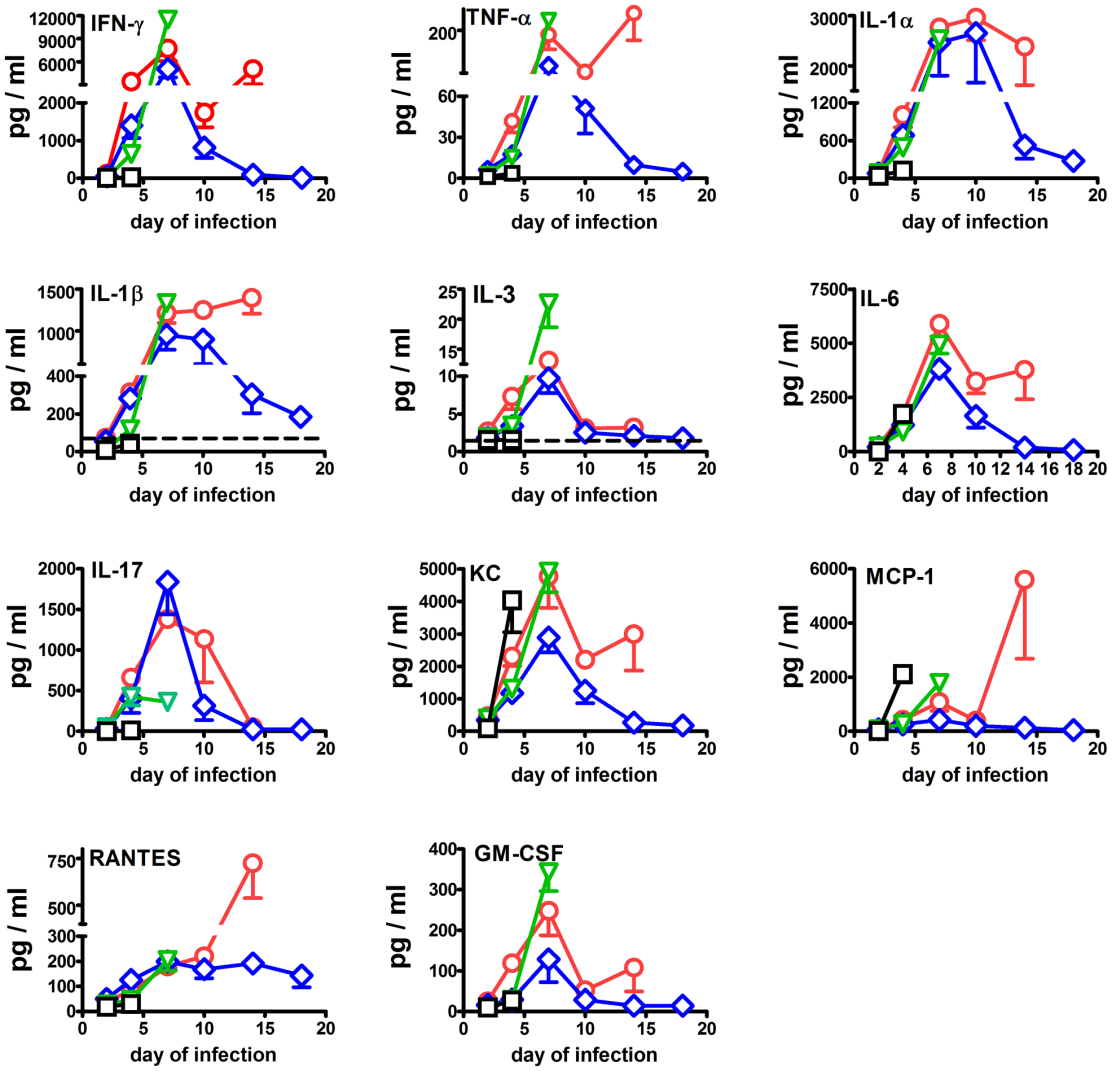
Changes in pulmonary cytokine and chemokine levels in naïve mice (black) and mice immunized ID with 10^5^ CFU of LVS (green), *ΔclpB* (blue), or *ΔfupAΔcapB* (red) then challenged by aerosol six weeks later with SCHU S4. Dashed horizontal line  =  mean background level.

Changes in the levels of 21 selected cytokines and chemokines were monitored in the immunized and challenged mice shown in figure 1. Differences between comparison groups were considered to be significant at P<0.05 or P<0.0045 as appropriate. The most pronounced changes in the lungs are shown in figure 3. By day 2 of infection, significant fold increases above background in the mean levels of IL-6 (40–55-fold), IL-17 (9–14-fold), KC (8–11-fold), MCP-1 (10–14-fold), and RANTES (2–4-fold) were observed in lung homogenates from all vaccinated groups, but not unvaccinated control mice. Additionally, levels of IFNγ (8–19-fold) and TNFα (3-fold) were significantly above normal in mice immunized with *ΔfupAΔcapB* or *ΔclpB* at this time. IL-1β (9–10-fold) levels were significantly depressed in naïve and LVS-immunized mice by day 2 of infection.

By day 4 of infection, levels of IL-6 (180–345-fold), KC (28–98-fold), MCP-1 (35–280-fold), and RANTES (2–9-fold) were significantly above background in all challenge groups. IFNγ (102–540-fold) was only elevated in the immunized groups. Between the infected groups MCP-1 (280-fold versus 35–55-fold) and KC (98-fold versus 28–55-fold) levels were significantly higher in the control versus any of the immunized groups at this time. In contrast, IFNγ (102–540-fold versus 3-fold), IL-17 (110–180-fold versus 3-fold), IL-1α (10–20-fold versus 3-fold), RANTES (3–9-fold versus 2-fold), and TNFα (8–23-fold versus 2-fold) levels were significantly higher in immunized versus control mice. Among the immunized groups, RANTES levels were significantly greater in mice immunized with *ΔclpB* versus the other vaccine strains (9-fold versus 3–6-fold), TNFα levels were significantly greater in mice immunized with *ΔfupAΔcapB* (23-fold versus 8–9-fold). GM-CSF (11-fold) and IL-3 (4-fold) levels were significantly higher than normal only in mice immunized with *ΔfupAΔcapB*. IL-1β levels were significantly increased (4-fold) only in mice immunized with *ΔclpB* or *ΔfupAΔcapB*.

All control mice were dead by day 7 of infection, by which time all three surviving vaccinated groups showed a further increase over background in levels of IFNγ (3000–6800-fold), TNFα (55–105-fold), IL-1α (65–72-fold), IL-1β (10–14-fold), IL-3 (4–9-fold), IL-6 (1200–1900-fold), KC (91–156-fold), MCP-1 (71–296-fold), RANTES (20-fold), and GM-CSF (9–23-fold). In contrast, IL-17 levels rose in mice immunized with *ΔfupAΔcapB* (355-fold above background) or *ΔclpB* (475-fold above background), but fell in mice immunized with LVS (94-fold above background). Levels of IL-12p40 were significantly depressed (5-fold) in LVS-immunized mice at this time, but were at normal levels in the other two groups of vaccinated mice, and hence do not provide a useful correlate of protection. No LVS-immunized mice survived to day 10 of infection. By this time most cytokines and chemokines had fallen markedly from their day 7 peaks, but remained significantly above background levels in the two surviving groups of mice. Notable exceptions were IL-1α, IL-1β and RANTES which remained at similar levels to day 7. Between the two surviving groups, there were no significant differences in the levels of any cytokine or chemokine at this time. By day 14 of infection, most cytokines and chemokines were declining towards background in surviving mice immunized with *ΔclpB*, whereas the opposite trend was observed for mice immunized with *ΔfupAΔcapB*. In particular, levels of IFNγ, IL-1α, Il-1β, IL-6, KC, MCP-1, MIP-1β, RANTES, and TNFα were significantly greater in mice immunized with *ΔfupAΔcapB versus ΔclpB* at this time. On the other hand, IL-1α, IL-3,and IL-17 levels declined in both groups between days 10 and 14. By day 18, IL-1α (7-fold), IL-6 (24-fold), KC (6-fold), MCP-1 (7-fold), and RANTES (15-fold) remained significantly elevated in the surviving *ΔclpB*-vaccinated mice.

In the lung, the majority of the cytokines were strongly correlated with bacterial load (Table 1). This suggests that cytokine levels, at least at a relatively early stage of infection (4–7 days), are affected by the bacterial burden. In an attempt to determine the effect of this variable on the molecular immune responses elicited by the three vaccine strains, the cytokine levels were modeled using ANOVA with bacterial load and immunization type as explanatory variables.

**Table 1:** The influence of immunization with *ΔfupAΔcapB (A)*, *ΔclpB (B)* or *LVS (C)* on pulmonary cytokine levels following aerosol challenge with SCHU S4.

		ANOVA p-values		Pair wise comparisons p-values		
Cytokine	Cor	Imm	CFU	A vs. B	A vs. C	B vs. C
GM-CSF	0,86	0,0328	0,0000	0,2519	0,0090	0,1134
IFNγ	0,84*	0,0013*	0,0000*	0,1871*	0,0092*	0,0004**
IL-10	0,56*	0,0004*	0,0004*	0,0761*	0,0012**	0,0001**
IL-12p40	−0,11*	0,0000*	0.2413*	0,0031**	0,0071*	0,0000**
IL-12p70	−0,54	0,2886	0,0579	0,1074	0,5603	0,2055
IL-17	0,20	0,0116	0.1465	0,5017	0,0095	0,0042**
IL-1α	0,76*	0,0005*	0,0000*	0,0344*	0,0103*	0,0000**
IL-1β	0,74*	0,0003*	0,0000*	0,0076*	0,0152*	0,0000**
IL-2	0,67*	0,0000*	0,0000*	0,5724*	0,0001**	0,0000**
IL-3	0,90	0,0516	0,0000	0,2469	0,0923	0,0116
IL-4	0,80	0,8098	0,0000	0,4194	0,7683	0,7010
IL-6	0,72	0,0035	0,0000	0,1812	0,0216	0,0001**
IL-9	0,37	0,0453	0.0343	0,1902	0,0171	0,1712
KC	0,89*	0,0015*	0,0000*	0,9676*	0,0014**	0,0039**
MCP-1	0,88	0,8857	0,0000	0,9629	0,6425	0,6721
MIP-1β	0,90	0,7349	0,0000	0,2139	0,9533	0,5242
RANTES	0,55*	0,0004*	0,0049*	0,0004**	0,0887*	0,0001**
TNFα	0,86*	0,0005*	0,0000*	0,0536*	0,0078*	0,0001**
VEGF	−0,90	0,1272	0,0000	0,0360	0,4712	0,1007

The analysis is based on lung data from day 4 and 7. *T*he correlation between cytokine levels and bacterial load are presented in column 1. The cytokine levels were modeled using ANOVA with the bacterial load (CFU) and the immunization (Imm) as explanatory variables; their corresponding *P* values are presented in columns 2 and 3. Cytokines significantly affected by the vaccines are highlighted by a single asterisk. The mean effects, after removing the affect of the bacterial load, are presented in columns 4–6. *P* values for the pair-wise comparisons are presented in columns 7–9 and significant findings are highlighted by a double asterisk.

For the cytokines IFN-γ, IL-10, IL-12-p40, IL-1α, IL-1β, IL-2, KC, RANTES and TNFα, the effect of the immunizing strain was significant. The *ΔclpB* mutant had the highest cytokine levels (after removing the effect of the bacterial load) followed by *ΔfupAΔcapB* and LVS. The difference between *ΔclpB* and *ΔfupAΔcapB* was significant for IL-12-p40 and RANTES, the difference between *ΔclpB* and LVS was significant for all the selected cytokines and the difference between *ΔfupAΔcapB* and LVS was significant for IL-10, IL-2 and KC. In addition, pair wise analysis on IL-17 and IL-6 were made and significant differences between *ΔclpB* and LVS were observed. The complete results are presented in Table 1.

### Changes in spleen cytokine and chemokine levels post-challenge

**Figure 4:**
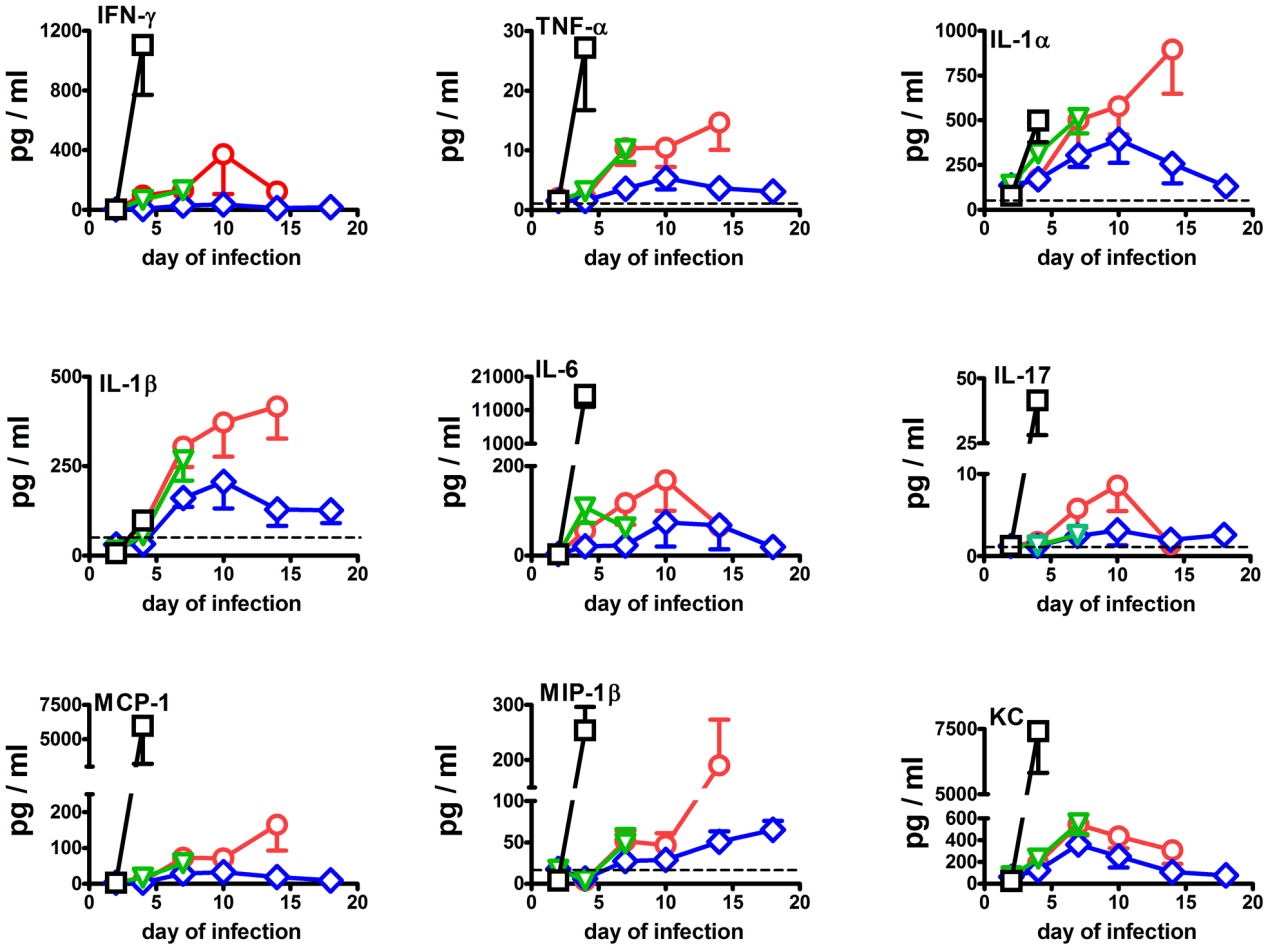
Changes in splenic cytokine and chemokine levels in naïve mice (black) and mice immunized ID with 10^5^ CFU of LVS (green), *ΔclpB* (blue), or *ΔfupAΔcapB* (red) then challenged by aerosol six weeks later with SCHU S4. Dashed horizontal line  =  mean background level.

The most pronounced changes in cytokine and chemokine levels in the spleen are shown in figure 4. In the spleen by day 2 of infection, KC levels were significantly (P<0.05) above background (3-fold) in immunized mice only. IL-1β levels were significantly depressed (3–6-fold) in mice immunized with LVS and naïve mice at this time. By day 4, levels of IL-1α (2.5–7.5-fold), IL-6 (5–4000-fold), and KC (6–365-fold) were significantly above normal in all four groups. IFNγ was significantly above normal in naïve mice (550-fold), and mice immunized with *ΔfupAΔcapB* (48-fold), or LVS (33-fold), but not in mice immunized with *ΔclpB* (3-fold); the levels in naïve mice were significantly higher than in any of the immunized mice. Levels of IL-6 (4000-fold versus 5–28-fold), and KC (365-fold versus 6–11-fold) were also significantly higher in naïve mice than in immunized mice at this time. Moreover, IL-17 (33-fold), MCP-1 (1830-fold), MIP-1β (15-fold), and TNFα (17-fold) were only significantly above background in naïve mice at this time. Amongst the immunized groups, IFNγ levels were significantly higher in LVS- (33-fold) and *ΔfupAΔcapB*- (48-fold) than in *ΔclpB*- (3-fold) immunized mice, and IL-6 levels were significantly higher in LVS- (28-fold) versus *ΔclpB*- (5.5-fold) immunized mice. By day 7, levels of IL-1α (4–7-fold), IL-6 (17–90-fold) and KC (20–30-fold) were significantly elevated in all three groups of vaccinated mice whereas, IL-1β (3–4-fold), IFNγ (26-fold), TNFα (5-fold), MIP-1β (2-fold), and MCP-1 (5–6-fold) levels were only elevated in mice immunized with LVS or *ΔfupAΔcapB*, and only the latter group exhibited an increase in IL-17 (5-fold). By day 10, IL-1α (6–8-fold), IL-6 (56–128-fold), KC (13–23-fold), were significantly elevated in both groups of surviving mice, whereas IL-1β (5-fold), IFNγ (75-fold), IL-17 (8-fold), TNFα (5-fold), MCP-1 (6-fold), and MIP-1β (2-fold) levels were only significantly elevated in mice immunized with *ΔfupAΔcapB*. By day 14, IL-1α (4–13-fold), IL-6 (50-fold), KC (6–17-fold) levels were significantly elevated in both surviving vaccine groups, whereas IFNγ (25-fold), IL-1β (5-fold), MIP-1β (9-fold), TNFα (7-fold), MCP-1 (15-fold), were only elevated in mice immunized with *ΔfupAΔcapB*. By day 18, IL-6 (15-fold) and KC (4-fold), were significantly above background in surviving *ΔclpB-* immunized mice.

### Changes in serum cytokine and chemokine levels post-challenge

**Figure 5:**
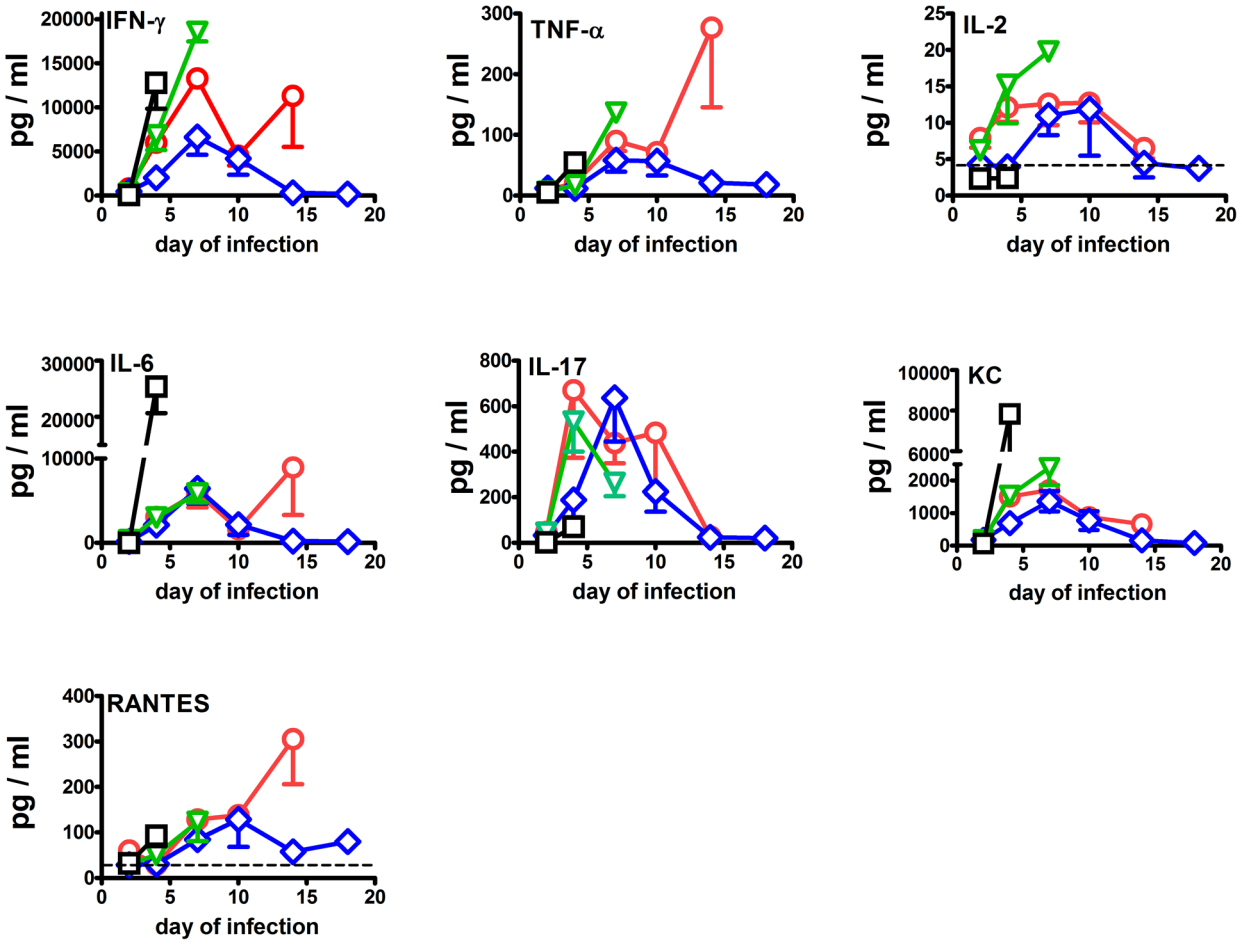
Changes in serum cytokine and chemokine levels in naïve mice (black) and mice immunized ID with 10^5^ CFU of LVS (green), *clpB* (blue), or *ΔfupAΔcapB* (red) then challenged by aerosol six weeks later with SCHU S4. Dashed horizontal line  =  mean background level.

The most pronounced changes in serum cytokine and chemokine levels are shown in figure 5. Compared to sera from unchallenged control mice, serum levels of IFNγ (mean 7–21-fold increase), IL-6 (20–40-fold increase), and IL-17 (mean 8–12—fold increase) levels were significantly raised in immunized, but not un-immunized mice on day 2 of infection. Additionally, serum IL-2 levels were significantly above normal in mice immunized with *ΔfupAΔcapB* (3-fold) at this time. By day 4, there was a further significant increase over background levels of IFNγ (50–180-fold), IL-6 (280–410-fold), and IL-17 (40–165-fold) in all three vaccinated groups. However, by this time the mean change in the level of IFNγ (335-fold) and IL-6 (3,400-fold) was greater in naïve *versus* any of the vaccinated groups; this difference was statistically significant for IL-6. IL-17 levels were also elevated in naïve mice at this time (17-fold), but not by as much as in vaccinated mice. KC levels were significantly above normal in all groups at this time (mean 10–125-fold). As at day 2 of infection, IL-2 levels were significantly higher than normal in mice immunized with *ΔfupAΔcapB* (5-fold) as well as in mice immunized with LVS (6-fold). Furthermore, levels of IFNγ were significantly greater in these two groups than in mice immunized with *ΔclpB* at this time. TNFα (3–10-fold) levels were significantly above background in all groups except mice immunized with *ΔclpB* at this time. By day 7 of infection, levels of IFNγ (90–250-fold), IL-6 (360–390-fold), IL-17 (30–75-fold), KC (20–35-fold), TNFα (5–13-fold), and IL-2 (2–3.5 fold) were significantly elevated above background in all three vaccinated groups. By day 10 post-challenge, levels of IFNγ (57–61-fold), IL-6 (99–132-fold), IL-17 (27–59-fold), TNFα (5–7-fold) and KC (12-fold) remained significantly above normal in both remaining groups of vaccinated mice. By day 14, IL-6 remained significantly above normal in both groups of immunized mice (10–550-fold) whereas IFNγ (150-fold), KC (10-fold), RANTES (7-fold), and TNFα (26-fold) were significantly above normal only in mice immunized with *ΔfupAΔcapB*. By day 18, only IL-6 levels (8-fold) were significantly above background in surviving mice immunized with *ΔclpB*. We have previously reported the same pattern of responses for naïve BALB/c mice challenged by aerosol with SCHU S4 [22].

### A putative role for IL-17 in pulmonary protection

The above cytokine and chemokine analyses showed that IL-17 level in the lungs on day 7 post-challenge was the main immune mediator that correlated with the superior efficacy of the SCHU S4 mutants versus LVS (figure 3). To determine whether IL-17 might be contributing to enhanced protection, mice immunized with *ΔclpB*, then challenged IN 6 weeks later with 100 CFU of SCHU S4 were treated with two anti-IL-17 mabs. This challenge regimen was chosen because it is appeared to be close to the threshold of the protective immune response (figure 2), and hence most likely to reveal any detrimental effects. In a preliminary experiment treatment at 2 h, 3 days and 7 days of such mice with the anti-IL-17A mab from R&D Systems had no effect on survival (not shown). Therefore, in a similar experiment some mice were treated on days 4, 5, 6, post-challenge with a combination of this mab and another anti-IL-17A mab from eBiosciences, or the corresponding isotype controls, and some were untreated. In this case, treatment was administered during the period of onset of pulmonary IL-17 production (figure 3) in the hope that this would achieve maximal effect. On day 7 post-challenge 5 mice/group were killed and organ bacterial burdens and lung cytokine levels determined (Table 2). Mice treated with anti-IL-17 mabs had a mean 3-fold reduction in IL-17 levels and a mean 10-fold increase in lung bacterial burden compared to the control groups, but these differences were not statistically significant. Moreover, IL-17 levels were still 50-fold above background in the mice treated with anti-IL-17 mabs. In keeping with the increased bacterial burden, mice treated with anti-IL-17 mabs had ∼3-fold more IFNγ in their lungs than the control groups, but this difference was not statistically significant. Mice not killed on day 7 were monitored for survival for 28 days post-challenge, and 1/5 untreated mice and 1/5 mice treated with anti-IL-17 mabs died during this time.

**Table 2:** Effect of anti-IL-17 MAb treatment on infection in mice immunized with *ΔclpB*.

Vaccinated mice treatment	Log_10_ lung bacteria ± SEM	Lung IL-17 levels ± SEM (pg/ml)	Lung IFNγ levels pg/ml± SEM
anti-IL-17 mabs	6.1±0.58	80.3±27.8	3148±1326
isotype matched mabs	5.1±0.61	241.8±78.3	1261±960
Untreated	5.2±0.88	215.1±68.2	1081±612
Untreated and uninfected		1.5±0.3	1.8±0.003

Mice (n = 5/group) immunized intradermally with 10^5^ CFU of *ΔclpB* were challenged 6 weeks later IN with 100 CFU of SCHU S4. Antibodies were administered IP on days 4, 5, 6 post challenge.

### Histopathology of pulmonary tularemia in mice immunized with *ΔclpB* or LVS

**Figure 6:**
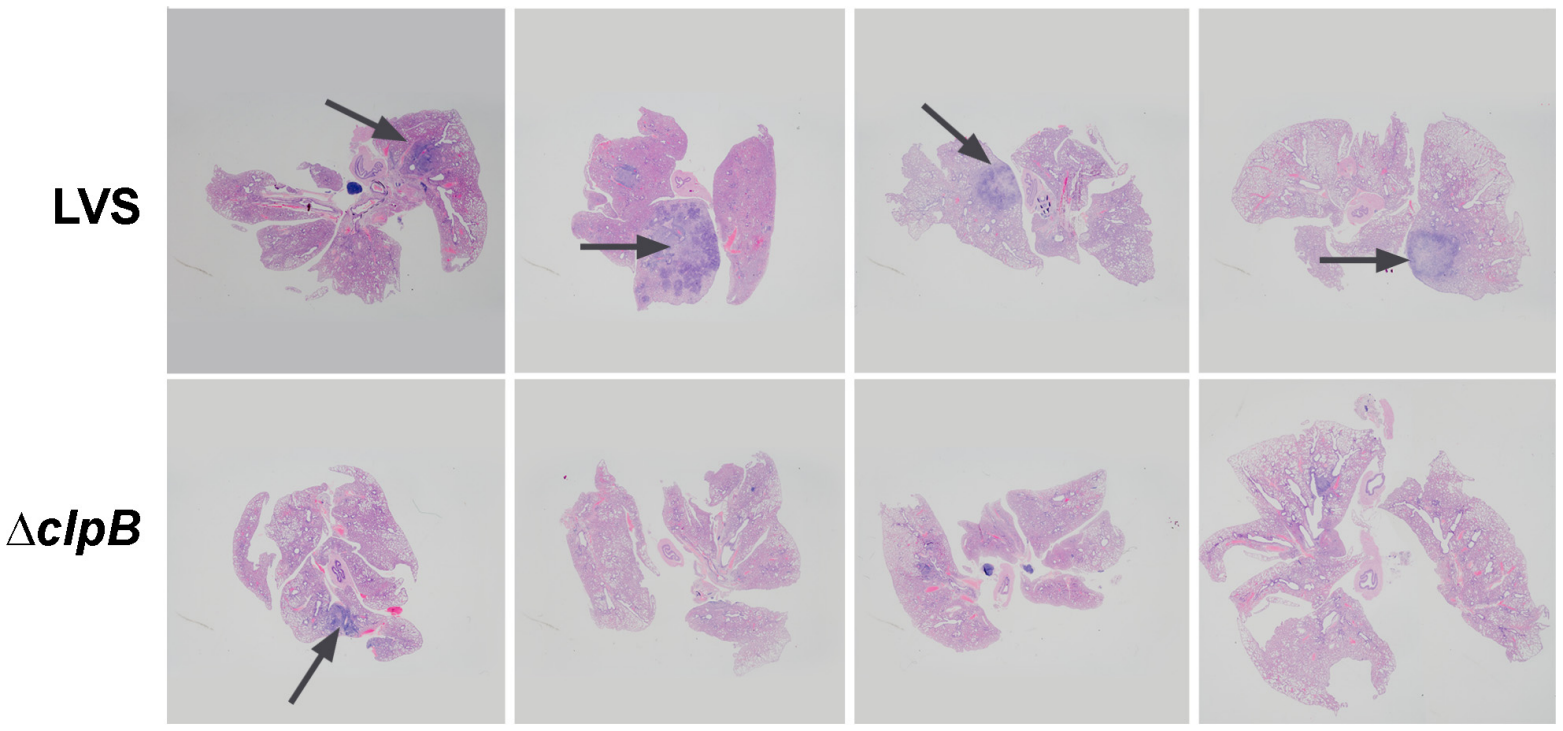
Histopathology of pulmonary tularemia in mice immunized with *ΔclpB* or LVS. Medium to large foci of lung consolidations (arrows) were seen in all LVS-immunized mice at day 7 post challenge, but only occasionally in *clpB*-immunized mice. Each photograph was taken from individual mice.

**Figure 7:**
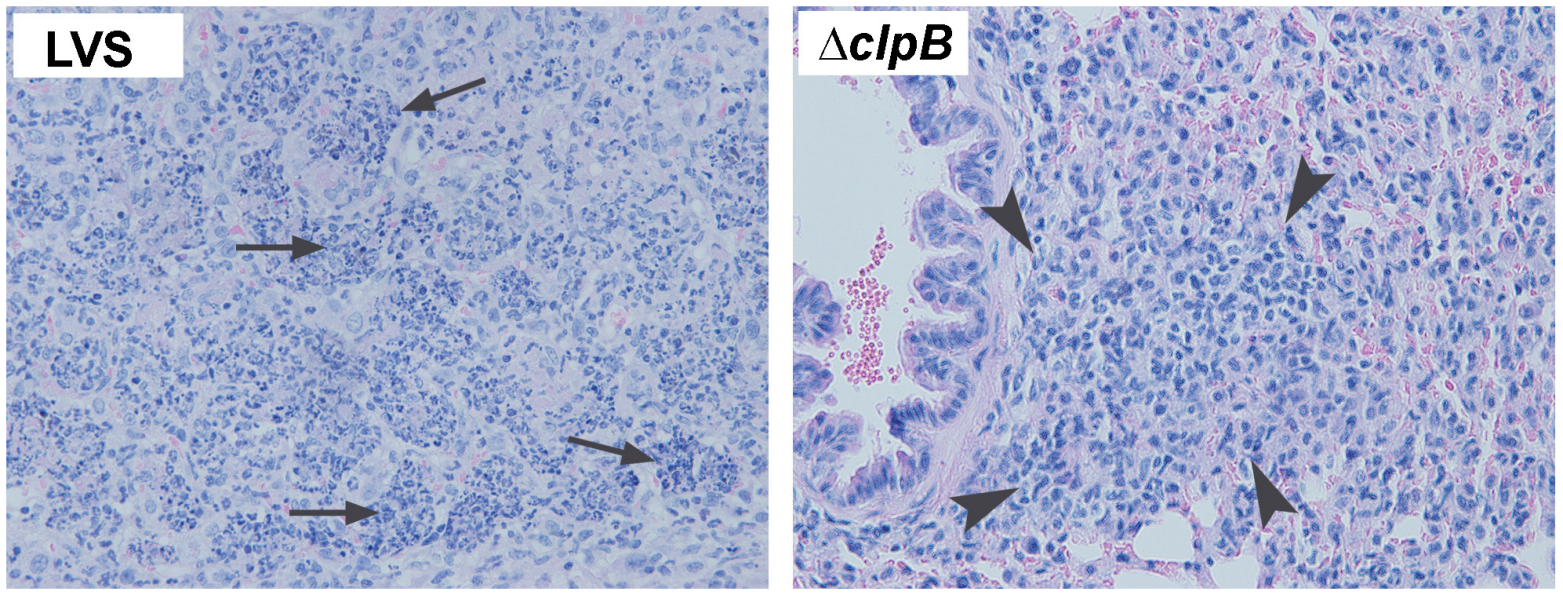
Histopathology of pulmonary tularemia in mice immunized with *ΔclpB* or LVS. **left**: The lung from an LVS-immunized mouse killed at day 7 post challenge showing severe subacute bronchopneumonia. The alveolar spaces are filled with large numbers of intact and degenerated neutrophils (arrows) admixed with small numbers of mononuclear cells. (**right**) the lung from a *clpB*-immunized mouse killed at day 7 post challenge showing the infiltration of predominantly mononuclear cells (arrowheads) with virtually no neutrophils in the peribronchial area. H&E, x400.

A histological examination of the lungs on day 7 post challenge showed medium to large foci of consolidations remain easily visible (Fig. 6) in the lungs from LVS-immunized mice and the alveolar spaces in these affected areas are filled with large numbers of intact and degenerated neutrophils admixed with small numbers of mononuclear cells (Fig. 7). In contrast, inflammation was less widespread in the lungs of the mice immunized with *ΔclpB* and consisted mainly of mononuclear cells.

## Discussion

The development of facile correlates of protection assays will facilitate the approval of tularemia vaccines for clinical use under the FDA Animal Rule. In mouse models of tularemia, highly attenuated LVS given ID protects against >1000 LD_50_ ID challenge with wild-type *F. tularensis* subsp. *tularensis*, but only prolongs life by a few days against a low dose aerosol challenge [8], [18]. A similar relative efficacy difference has also been shown for humans [2], [3]. More recently, Fischer rats immunized with LVS were shown to resist respiratory challenge with up to 10^8^ CFU of SCHU S4 [23].This appears to be a 10,000-fold higher challenge dose than the breakthrough dose for LVS-immunized humans [4]. Thus, it appears that LVS-elicited protection is exaggerated in rats, but understated in mice, compared to humans. It is possible that vaccines that perform better than LVS in mice will also do so in humans. In contrast, it seems unlikely that anything can be shown to be substantially superior to LVS in the Fischer rat model. Recently, we showed that deleting either the heat shock gene, *clpB*, alone, or the iron-acquisition gene, *fupA*, and putative capsule gene, *capB*, together, created highly attenuated mutant strains of *F. tularensis* subsp. *tularensis* strain SCHU S4. When administered ID to BALB/c mice the SCHU S4 deletion mutants provided better protection than LVS against a subsequent low dose aerosol challenge with the wild-type strain [21]. This finding was confirmed in the present study and was extended by showing that mice immunized with *ΔclpB* were significantly better protected than LVS-immunized mice following respiratory challenge with up to 10^3^ CFU of SCHU S4.

Additionally, herein we have compared global molecular immune responses in mice vaccinated with one or other attenuated strain and challenged with an aerosol of SCHU S4 in an attempt to establish a correlate of protection. Several cytokines were found at higher levels in the lungs of immunized versus naïve mice, but amongst the immunized groups, there was little direct correlation between pulmonary cytokine levels and protective efficacy. Instead, they mostly rose in response to increasing bacterial burden.

Amongst all of the pulmonary cytokines and chemokines examined, peak levels of pulmonary IL-17 that occurred on day 7 of infection provided the most straightforward potential correlate of protection. By this time, significantly greater levels (4–5-fold) of IL-17 were found in the lungs of mice immunized with either of the SCHU S4-based vaccines versus LVS, despite the fact that pathogen burden in this organ was 10-fold greater in the latter versus former groups. Interestingly, reducing IL-17 levels in the lungs of mice immunized with *ΔclpB* by 3-fold, also caused a 10-fold increase in bacterial burden by day 7 of infection. Additionally, IL-12p40 levels were significantly depressed in LVS-immunized mice at this time, and IL-12p40 levels have been shown to be co-dependent on IL-17 levels during primary pulmonary infection of mice with LVS[24], [25]. However, as unadjusted pulmonary IL-12p40 levels bacteria remained at normal levels in mice immunized with the SCHU S4 mutants, this cannot function as a straightforward correlate of protection.

When adjusted for the effects of the different bacterial burdens, several additional cytokines and chemokines were present at relatively higher levels in the lungs of the mice immunized with *ΔclpB*, and to a lesser extent, in the lungs of mice immunized with *ΔfupAΔcapB*, than in the lungs of mice immunized with LVS. This could help explain the apparent paradox of having greater total amounts of IFN-γ in the lungs of the mice immunized with LVS versus the other two vaccine strains on day 7 of infection, given that this cytokine is critical for the expression of anti-*Francisella* immunity.

After day 7, IL-17 levels declined in the surviving mice immunized with either of the SCHU S4 mutants even as bacterial burden rose in the lungs of mice immunized with *ΔfupAΔcapB*. In contrast, pulmonary IFN-γ levels rose in the latter mice in response to recrudescent infection. This suggests that IL-17 and IFNγ arose from different sources or were under different feedback control.

Many of the same cytokine and chemokine expression patterns were observed in the spleens of immunized mice, but were generally more muted than in the lungs, particularly in mice immunized with *ΔclpB*. However, there were approximately 100-fold fewer bacteria in the spleens versus the lungs on any given day of infection. In this light, relative amounts of some cytokines and chemokines were similar or higher in the former organ, especially in naïve mice. In particular, this further emphasizes the presence of high levels of IFNγ, TNFα, IL-1α, IL-17, and MIP-1β, evident in the spleens versus the lungs of naïve infected mice. This finding is in keeping with that of others showing that pulmonary immune responses are suppressed in naïve mice challenged by aerosol with SCHU S4 [26] On the other hand, KC levels were highest in the lungs of naïve mice on day 4 of infection suggesting some degree of selectivity to the pulmonary immunosuppression.

Changes in absolute cytokine and chemokine levels in the serum did not fully reflect the changes observed in the lungs or spleen. In particular, the dramatic increases in IL-1α levels seen in the lungs, and to a lesser extent in the spleens, were not observed in the serum. Relative differences in serum IL-17 levels among the immunized groups were the same as those observed in the lungs on day 7, but were less obvious. Moreover, there was a temporal component to this, since mice immunized with *ΔclpB* had significantly lower levels of serum IL-17 than the two other vaccinated groups on day 4 of infection. On the other hand, the increases observed in the spleens, but not the lungs of naïve mice in IFNγ, IL-6, and TNFα, were recapitulated in the serum. Because bacteremia was not universally present, it was not possible to determine the effect of this variable on cytokine and chemokine levels in this tissue. Levels of most cytokines and chemokines, remained close to or substantially below background levels in liver homogenates in this study, suggesting an assay interference problem.

Although bacterial burden adjustments demonstrated that relative levels of several pulmonary cytokines and chemokines were higher in mice immunized with the most protective vaccine strain versus LVS, such adjustments cannot be extended to humans. Thus, for practical purposes, only biomarkers that increase in absolute amounts during a protective immune response can be used as potential correlates of protection. In this regard, enhanced absolute IL-17 levels in the lungs on day 7 of infection was the immune mediator that clearly distinguished between mice immunized with SCHU S4 mutants versus more poorly protected mice immunized with LVS.

Several other groups have recently shown that IL-17 is critical for controlling primary pulmonary infection of mice with LVS [24], [25], [27]. They have shown too that multiple types of leukocytes, including various T cell subsets, produce this cytokine in the lungs during primary infection. In particular, CD4^+^, CD8^+^ and CD4^−^CD8^−^ T cells induced by IN vaccination were all able to activate macrophages *in vitro* to kill ingested LVS [24]. Moreover, this immune response is not elicited by ID vaccination with LVS [24], [28]. Given that pulmonary vaccination with LVS generates better protection than ID vaccination against pulmonary challenge with clinical strains of subsp. *tularensis*, it is interesting to speculate whether this is due to the induction of IL-17 producing T cells by the former regimen. However, there are a number of caveats to consider that could render moot such speculation. Firstly, all of the aforementioned studies with highly attenuated LVS used C57BL/6 mice that we and others have been unable to protect against subsp. *tularensis* challenge [8], [18], [19]. Secondly, it is not known whether the aforementioned immune responses persist beyond the resolution of primary infection. Thirdly, the importance of a particular immune response for combating primary infection does not predict its requirement for alleviating secondary infection. For instance, neutrophils, TNFα and IFNγ are far more important for controlling primary versus secondary LVS infection [29], [30]. Fourthly, the importance of a particular host defense in protection against LVS does not predict its importance in protection against more virulent strains. For instance, depleting immunocompetent mice of TNFα, IFNγ, or neutrophils renders them much more susceptible to infection with LVS, but not to virulent subsp. *holarctica* or *tularensis* [31]. Finally, the current study deals with intradermally vaccinated mice that do not elicit an obvious IL-17 response in the lungs unless subsequently challenged with SCHU S4. Given these provisos it is clear that a systematic examination of the cells involved in the control of SCHU S4 infection in the lungs of BALB/c mice after ID vaccination with *ΔclpB* versus LVS will be required to determine the extent to which they overlap with cells required to control primary lung infection with attenuated LVS in C57BL/6 mice. This work is beyond the scope of the current study.

IL-17 has been shown to participate in host defense against primary infection with LVS and other intracellular bacterial pathogens [24], [25], [27], [32], [33], and in vaccine-elicited acquired immunity against tuberculosis [34]. Thus, it would be surprising if this cytokine did not contribute to some degree to vaccine-elicited immunity to inhaled *F, tularensis* regardless of its cellular source. There are a variety of ways by which IL-17 could contribute to protection against *F. tularensis*. For example, IL-17 promotes the production of antimicrobial proteins in the lungs [35] and the infiltration of other leucocyte populations in this organ including IFNγ-secreting Th1 cells [34] that are known to be important for combating inhaled *F. tularensis*. However, the histological data presented herein clearly shows that the enhanced levels of IL-17 in the lungs of mice immunized with *ΔclpB* versus LVS does not result in an overall increase in cellular inflammation. Indeed, in keeping with the lower levels of other proinflammatory mediators in the lungs such as IL-6 and KC, overt pulmonary inflammation, especially infiltrating neutrophils is reduced in the lungs of mice immunized with *ΔclpB* versus *LVS* following respiratory challenge. It remains to be determined why immunization with the SCHU S4-based mutants resulted in higher peak levels of IL-17 in the lungs. This might be revealed by an examination of the cell-mediated immune responses to vaccination alone without subsequent challenge, and such studies are planned.

Regardless, of its contribution to anti-*Francisella* immunity, absolute changes in IL-17 levels in the sera of immunized and challenged mice were not sufficient to predict relative efficacy of the test vaccines with a high degree of confidence which would seem to rule it out as a target for developing a clinically facile correlate of protection. Others recently showed that peripheral blood CD4^+^ and CD8^+^ T cells from humans immunized with LVS produced IL-17 upon re-stimulation with a whole cell lysate of LVS [36]. However, since these individuals were not challenged with virulent bacteria, and since not all LVS-vaccinated humans are protected post-immunization [2], [3] it is not possible to determine whether IL-17 served as a correlate of protection in this situation.

## Materials and Methods

### Ethics Statement

Mice were maintained and used in accordance with the recommendations of the Canadian Council on Animal Care Guide to the Care and Use of Experimental Animals. The work was approved by the Animal Care Committee of the National Research Council, Institute for Biological Sciences under protocol number 2008-06.

### Mice

Female BALB/c mice were purchased from Charles Rivers Laboratories (St. Constant, Quebec) and entered experiments at 8–10 weeks of age.

### Bacteria

The ATCC isolate 29684 of LVS was used for comparison with SCHU S4-based vaccines. Wild-type SCHU S4 was obtained from the Francisella Strain Collection maintained at Umea University, Sweden. Mutants containing in-frame deletions of the *clpB* and *capB* genes, respectively, of SCHU S4 have been previously described [21]. The relative efficacies of LVS vs these two mutants against aerosol challenge with SCHU S4 have been described previously [21]. For the present study, stock cultures of all strains were prepared by growing them as confluent lawns on cystine heart agar supplemented with 1% (w/v) hemoglobin (CHAH). Bacteria were harvested after 48 h incubation at 37°C into freezing medium consisting of modified Mueller Hinton broth containing 10% w/v sucrose. Stocks were aliquoted in a volume of 1 ml and stored at −80°C at a concentration of 10^10^–10^11^ CFU/ml.

### Immunization and challenge

Based on previous findings we chose 10^5^ CFU as the ID immunizing dose for all of the test vaccine strains in the current study. ID inocula were injected into a fold of skin in the mid-belly in a volume of 0.05 ml saline. Mice were challenged six-weeks post-vaccination with SCHU S4. Aerosol challenges were performed using an InTox Products nose-only exposure chamber as previously described [7], [18]. The protocol results in the delivery of ∼20 CFU of SCHU S4 to the lower airways of mice. Intranasal (IN) challenge was performed on anesthetized mice (10 µl of inoculum was administered to each nare followed by an equal volume of saline). All animal work was performed in a federally-licensed and Select-Agent-approved small animal containment level 3 facility. Mice were examined daily for signs of infection and whenever feasible were euthanized by CO_2_ asphyxiation as soon as they displayed signs of irreversible morbidity. Rat anti-mouse IL-17 monoclonal antibodies (mab) and isotype matched control antibodies were purchased from R&D Systems (catalog numbers MAB421 and MAB006), Minneapolis, MN and eBiosciences, San Diego, CA (catalog numbers 16-7173-85 and 16-4714-85). Immunized mice received 100 µg of each anti-IL-17 mab, or each control mab IP on days 4,5,6 post-challenge. Used singly, both antibodies have been shown to partially abrogate immune responses in a variety of disease models [37], [38], [39], [40].

### Bacteriology and cytokine assays

On the stated days post-challenge, some mice (n = 4/group) were killed and blood for serum was collected by cardiac puncture. For bacteriology, an aliquot of whole blood was diluted in nine volumes of sterile water to lyse host cells. The lungs, spleens, and livers were removed and homogenized in the presence of protease inhibitors (Complete Protease Inhibitor Cocktail Tablets, Roche Applied Sciences, Laval, Quebec), then placed on ice. Homogenates were diluted in saline and plated on CHAH agar. Thereafter, homogenates were clarified by centrifugation and the supernatants were filtered through a 0.22 µm membrane and then plated to confirm their sterility. Serum was similarly sterilized, and all samples were stored frozen at −20°C until needed. Preliminary studies demonstrated that the protease inhibitors had no effect on the recovery of viable bacteria. Levels of cytokines and chemokines were determined using Beadlyte® Mouse 21-plex Cytokine Detection System on a Luminex® 100 IS system (Luminex, Austin, TX). Serum samples (25 µl) were diluted 1∶2 and organ homogenates were used neat and analyzed as specified by the manufacturer. The cytokine/chemokine concentrations were calculated against the standards using Beadview® software version 1.03 (Upstate).

### Histology

Lungs were harvested *en bloc* into 10% buffered formalin and processed by standard pafaffin embedding techniques. Sections were cut at 4 µm, stained with hematoxylin and eosin and examined by light microscopy.

### Statistical analysis

Differences among the groups in the levels of bacteria and cytokines and chemokines were separately compared statistically by ANOVA with Dunnett's or Tukey's post-hoc test, or by t test as appropriate on log transformed data using GraphPad Prizm® version 4.0 software. Differences were considered to be statistically significant when P<0.05.

The above analyses do not take into account that cytokines levels may be directly affected by the levels of bacteria. Therefore, an additional complementing analysis was performed. Here the cytokine levels (log_10_-transformed) were modeled using an ANOVA with bacterial load (log_10_ CFU) and vaccine type (LVS, ΔclpB mutant or ΔfupAΔcapB mutant) as explanatory variables. For this purpose we used data from lung samples collected on day 4 and 7. This model allows us to compare the effects of the immunizations after that the influence of the bacterial load has been removed. Data from 19 cytokines were modeled (IL-13 and IL-5 were not modeled due to low quality data). The immunizations that had a statistically significant (Bonferroni corrected P<0.05, i.e. P<0.05/19) effect on the cytokines levels were studied further by performing pair-wise differences between the immunizations. The estimated affect of the bacterial load were removed from the cytokines levels and the residuals were used to compare all pairs of immunizations using Welch's t test. Pair-wise differences were considered to be statistically significant when the Bonferroni corrected P<0.05, i.e. P<0.05/11.
